# FeCl_3_-Intercalated Carbon Nanotube Film for Long-Term Infrared Camouflage in Harsh Environments

**DOI:** 10.3390/mi17010038

**Published:** 2025-12-29

**Authors:** Yijie Li, Zixuan Wang, Yong Wang, Ruiyun Chen, Ganying Zeng

**Affiliations:** 1 State Key Laboratory of Quantum Optics Technologies and Devices, Institute of Laser Spectroscopy, Shanxi University, Taiyuan 030006, China; 202322607030@email.sxu.edu.cn (Y.L.); 202322607074@sxu.edu.cn (Z.W.); 15835119755@163.com (Y.W.); chenry@sxu.edu.cn (R.C.); 2 Collaborative Innovation Center of Extreme Optics, Shanxi University, Taiyuan 030006, China

**Keywords:** carbon nanotube, FeCl_3_ intercalation, Fermi energy, charge transfer, infrared camouflage

## Abstract

Infrared camouflage, realized by engineering temperature and spectral emission characteristics, is crucial in various scientific and engineering fields. Yet, a significant challenge lies in fabricating advanced functional materials that can durably maintain infrared camouflage performance under harsh operational conditions. Herein, we report a FeCl_3_-intercalated carbon nanotube (CNT) film fabricated via a vapor intercalation strategy, with FeCl_3_ molecules inserted into the interlayer spacing of the CNT. Compared with pristine CNT, the FeCl_3_-intercalated CNT composite demonstrates significantly enhanced infrared camouflage capabilities, exhibiting apparent temperature variations of +16.7 °C, −6.6 °C, and −144 °C relative to the CNT film, under low (−4 °C), body (34.3 °C), and high (300 °C) temperature backgrounds, respectively. Moreover, extensive durability tests involving heat, insolation, and rain have confirmed the unaltered infrared camouflage performance of the FeCl_3_-CNT film. The performance enhancement is attributed to the suppressed infrared absorptivity across the 2.5–15.2 μm wavelength range, with a pronounced reduction from 72% to 30% at 15 μm, driven by intercalation-induced charge transfer and the consequent Fermi energy (E_F_) shift. This work presents a promising approach for designing advanced functional materials to achieve long-term infrared camouflage in complex environments.

## 1. Introduction

With the rapid advancement of military detection technologies, infrared camouflage materials and structural designs for effectively concealing military targets in complex environments have drawn extensive attention. Fundamentally, infrared camouflage is achieved by reducing the infrared radiation contrast between a target and its surrounding environment [1]. According to the Stefan–Boltzmann law, the two main approaches to increasing infrared camouflage are reducing emissivity and controlling temperature [2,3]. Notably, emissivity, as a unique material-dependent parameter, varies significantly with the microstructure and chemical composition of materials [4]. Conventional metallic material coatings such as Cu and Al exhibit superior infrared camouflage performance owing to their low emissivity [5]. Considerable oxidation tendency, excessive film weight, poor mechanical properties, and complex adhesive manufacturing processes have become the main constraints of the coatings [6,7]. Carbon-based materials are well-suited for designing infrared camouflage metal composites with enhanced stability due to their large specific surface area, lightweight, high mechanical strength, excellent electrical conductivity, and thermal stability [8]. For example, Zhang et al. investigated an aluminum and carbonized waste cotton–carbon felt composite fabric. Leveraging the low infrared emissivity of Al nanoparticles and the outstanding mechanical properties of hydrophobic flame-retardant aramid fabric, this composite presents good infrared camouflage capability [9]. Hassan fabricated a flexible Janus film integrating Al-rich Ti_3_C_2_T_x_ MXene and CNT film through hydrogen bonding, which shows an enhanced thermal camouflage performance [10,11]. The integration strategy enhances material stability to a certain extent, yet the intrinsic metallic components remain unable to fundamentally address the critical flaws that severely undermine the long-term reliability of infrared camouflage performance.

CNTs stand out in infrared optoelectronics due to the coupled influence of bandgap-mediated interband transitions and carrier-induced intraband absorption [12]. The chirality and diameter of CNTs control their bandgap structure, thereby governing whether these nanostructures function as metals or semiconductors [13,14]. Furthermore, carrier concentration can be modulated by external stimuli, including structural defect engineering [15], impurity doping [16], electric fields [17], and heterojunction construction [18]. Compared with these methods, intercalation enables distinctive modulation of light–matter interactions in two-dimensional materials by inserting ions, molecules, or metals into their van der Waals interlayer gaps, a process that alters interlayer coupling, electronic band structures, and surface charge distribution [19,20,21,22]. This approach has demonstrated diverse novel phenomena, such as superconductivity [23], ferromagnetism [24], charge density wave [25], color change [26], second harmonic generation [27], nonlinear absorption [28], and so on. To date, hundreds of chemical species that can be intercalated into carbon materials have been reported [29,30,31]. Compared with their counterparts (e.g., Li), FeCl_3_ molecules are larger in scale. Relevant research results show that the layer spacing of graphene increases to 9.4 Å after FeCl_3_ intercalation, but only 3.7 Å after Li intercalation [32,33,34,35]. Consequently, it induces a more significant structural transformation, thereby leading to alterations in the electronic band structures. Additionally, notable charge transfer happens at the interface between graphene layers and FeCl_3_ molecules, causing the E_F_ of graphene to shift from 0 to 0.9 eV upon FeCl_3_ intercalation [36]. Therefore, the combination of CNT as a host and FeCl_3_ as a guest may generate better advanced functional materials for infrared camouflage.

In this work, we successfully prepare FeCl_3_-intercalated CNT (FeCl_3_-CNT) with a vapor phase intercalation method. The technique exploits a temperature gradient between the FeCl_3_ precursor evaporation zone and the CNT reaction zone, thereby enabling controlled diffusion of FeCl_3_ vapor into the layer space of the CNT. An enhanced infrared camouflage of CNT film is observed after FeCl_3_ intercalation. Under low (−4 °C), body (34.3 °C), and high (300 °C) temperature backgrounds, FeCl_3_-CNT shows apparent temperature increases of 16.7 °C, and decreases of 6.6 °C and 144 °C, respectively. The infrared emission temperature of FeCl_3_-CNT demonstrates a tendency to follow the thermal fluctuations of the ambient air environment, thereby improving its infrared camouflage capability. Moreover, under various harsh environmental conditions such as high temperature, solar irradiation, and rainfall, the FeCl_3_-CNT film maintains consistent infrared stability without performance degradation. Our findings indicate that intercalation is an effective method to construct robust infrared camouflage materials.

## 2. Results and Discussion

**Figure 1:**
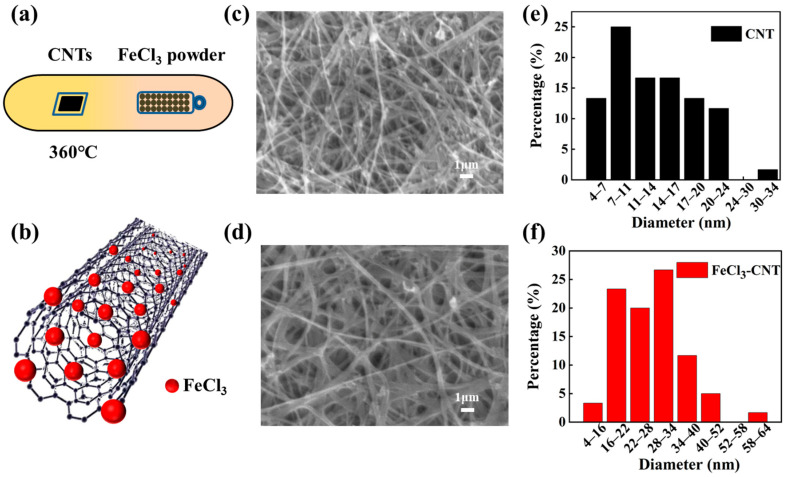
(**a**) Experimental setup of the dual-zone vapor transport method for FeCl_3_ intercalation into CNT, with CNT films contacting a quartz substrate. (**b**) Side-view schematic illustrating FeCl_3_ molecular intercalation within multi-walled carbon nanotubes. (**c**,**d**) SEM images of CNT before and after FeCl_3_ intercalation. (**e**,**f**) CNT diameter distribution before and after FeCl_3_ intercalation.

Figure 1 illustrates the sample fabrication process. As shown in Figure 1a, FeCl_3_-CNT is synthesized via a dual-zone vapor phase intercalation method. Anhydrous FeCl_3_ powder and CNT deposited on quartz substrates were positioned in separate zones within an argon-filled quartz tube. By exploiting the vapor pressure of FeCl_3_ at approximately 310 °C, FeCl_3_ molecules or FeCl_4_^−^ ions diffuse into the graphitic layers of the CNT [35]. This process is facilitated by the system’s inherent tendency toward lower energy, which arises from charge transfer interactions between the electron-accepting FeCl_3_ and the π-electron system of the graphene layers [37]. The structural configuration of FeCl_3_-CNT is schematically represented in Figure 1b, where each red sphere denotes a FeCl_3_ molecule. Pristine CNTs feature an interwoven network morphology, which is retained in FeCl_3_-CNT without significant structural degradation, as supported by SEM images in Figure 1c,d. The overall structural integrity of the CNT framework, with no obvious breaking, indicates its mechanical properties are well-maintained. The diameter distribution of the CNT is determined from 60 tubes, with each tube’s diameter calculated as the average of measurements from its different regions. As shown in Figure 1e,f, the peak of tube diameter distribution ranges from 7–11 nm in pristine CNT but 16–34 nm in FeCl_3_-CNT. This significant size expansion directly results from the intercalation of FeCl_3_ molecules into the interlayer spaces of the CNT [38].

**Figure 2:**
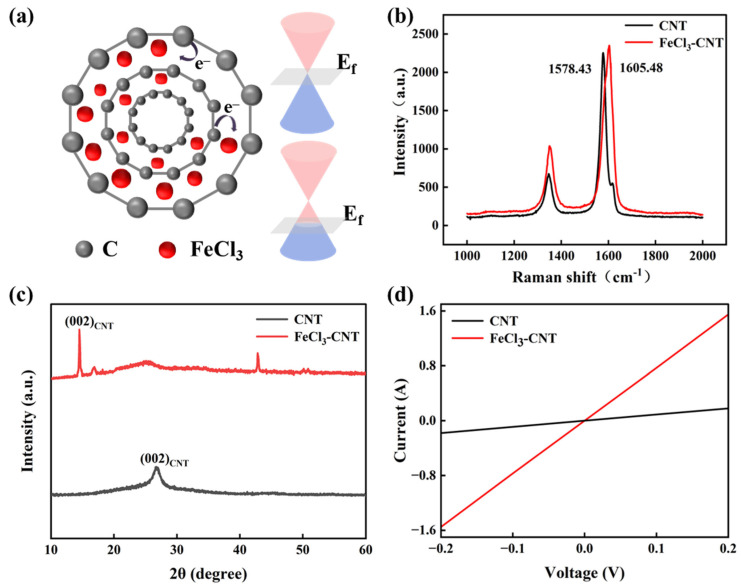
(**a**) Cross-sectional schematic of electron transfer during FeCl_3_ intercalation and corresponding Fermi energy shift. (**b**–**d**) show the Raman spectra, XRD, and four-point probe I−V curves, respectively. The black curves correspond to pristine CNT and the red curves to FeCl_3_-CNT.

The electronic redistribution mechanism is illustrated in Figure 2a. Upon FeCl_3_ intercalation, electron transfer from CNT to FeCl_3_ molecules induces pronounced p-type doping. This charge transfer effectively removes electrons from the CNT valence band, shifting the Fermi energy towards the valence band maximum (VBM) [39]. This shift in E_F_ fundamentally modifies the electronic structure near the Dirac point, increasing the density of holes as the primary charge carriers. Raman spectroscopy (Figure 2b) reveals critical structural modifications. The pristine CNT (black curve) exhibits characteristic peaks, with the G-band at 1578 cm^−1^ due to in-plane vibrations and 2D-band at 2710 cm^−1^. After intercalation (red curve), the G-band undergoes a 27 cm^−1^ blue shift to 1605 cm^−1^. This result aligns with intercalation stage 2, where each graphene sheet has only one adjacent FeCl_3_ layer [40]. This large blue shift is attributed to the phonon stiffening due to hole doping in the graphene layers of the CNT. Because the removal of electrons increases the force constants governing the C-C bond vibrations, leading to a higher frequency G-band mode. This observation provides spectroscopic evidence for the successful charge transfer from the CNT to the FeCl_3_ intercalant, consistent with the prior studies [41]. Neither pristine CNT nor FeCl_3_-CNT exhibits a significant D-band, suggesting that intercalation does not induce any structural defects.

XRD analysis demonstrates lattice expansion, as shown in Figure 2c. The (002) diffraction peak of pristine CNT at 26.7° shifts to 14.5° in FeCl_3_-CNT. By Bragg’s law (n λ = 2dsinθ) [42], the interlayer distance is calculated to expand from 0.334 nm to 0.6 nm, which is a structural consequence of accommodating the FeCl_3_ molecules (or ions) within the graphene interlayer of the CNT. This observation is indicative of intercalation stage 2 [43] and shows consistency with the Raman analysis results. Electrical characterization (Figure 2d) demonstrates a dramatic enhancement in conductivity after intercalation. The sheet resistance decreases sharply from 1.96 Ω/sq (pristine CNT) to an exceptionally low 0.12 Ω/sq (FeCl_3_-CNT). This improvement is primarily attributed to the significantly increased carrier density (holes) injected into the CNT framework via the FeCl_3_-derived p-type doping. The minimal structural disruption observed by SEM and the high D-band intensity in Raman suggest that this conductivity boost is achieved while largely preserving the intrinsic high carrier mobility of the CNT, indicating that the increased carrier density is the dominant factor rather than a reduction in scattering centers. This synergistic effect of high carrier density and maintained mobility is key to the remarkable electrical performance.

**Figure 3:**
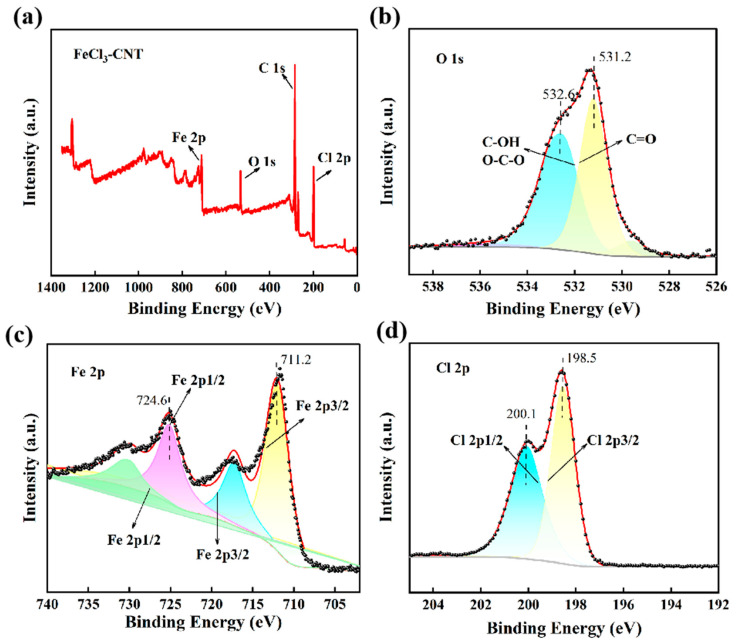
XPS analysis confirming the successful FeCl_3_ intercalation and p-type doping of CNT. (**a**) Survey spectrum showing the presence of C, Fe, O, and Cl. (**b**) O 1s spectrum. (**c**) Fe 2p spectrum. (**d**) Cl 2p spectrum.

XPS analysis confirms the successful FeCl_3_ intercalation into CNT bundles. The survey spectrum (Figure 3a) clearly shows the presence of C, Fe, O, and Cl, supporting the incorporation of FeCl_3_ into the CNT structure [44]. In the O 1s spectrum (Figure 3b), the main peak at 531.2 eV corresponds to lattice oxygen in an FeOCl-like phase, indicating limited surface oxidation [45], while the minor peak at 532.6 eV is attributed to adsorbed water or hydroxyl groups. The Cl 2p spectrum (Figure 3d) shows a Cl 2p_3/2_ peak at 198.5 eV, slightly lower than that of pristine FeCl_3_ [46]. This peak shift indicates an increased electron density around Cl atoms arising from charge transfer, serving as a key signature of p-type doping. The Fe 2p spectrum (Figure 3c) displays peaks at 711.2 eV and 724.6 eV, along with satellite features, confirming the presence of Fe^3+^ and the stability of the intercalated species. These results collectively verify FeCl_3_ intercalation and its role in modifying the electronic structure of CNT [47].

**Figure 4:**
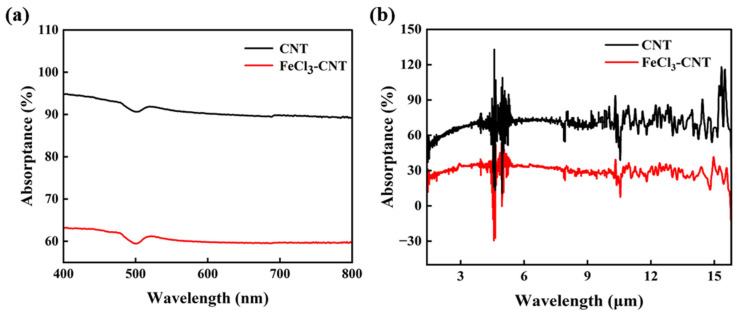
Wavelength-dependent absorptivity of pristine CNT (black) versus FeCl_3_-CNT (red) in (**a**) visible and (**b**) infrared spectral regions.

Intercalation-induced doping can affect the optical transition process, as proved by optical absorption spectra in the visible and infrared range. Figure 4a shows the absorption spectra in the range of 400–800 nm before and after FeCl_3_ intercalation. After intercalation, the broadband reduction occurs from ~95% to ~62% at 400 nm, ~92% to ~60% at 500 nm, and ~90% to ~60% at 700–800 nm. In the infrared range (Figure 4b), similar broadband suppression of optical absorption is caused by FeCl_3_ intercalation. For the pristine CNT film, infrared absorption remains at ~72% across the wavelength range of 4.3–15.7 μm, with a minimum of 51% at 1.6 μm. For FeCl_3_-CNT, the absorption reduces to ~30% across the wavelength range from 2.5 to 15.2 μm. The reduction in optical absorption can be accounted for as follows. Owing to the difference in electron gain/loss capabilities between the carbon layer and FeCl_3_ molecules, electron transfer occurs at their interface, as demonstrated by the Raman characterization and I–V curves analysis in Figure 2b,d. According to relative studies, this intercalation process ultimately renders the carbon material in a p-type doped state with a doping concentration of 1 × 10^15^ cm^−2^ and an E_F_ shift to ~0.9 eV in the valence band [48,49]. The optical conductivity of carbon materials can be characterized using the Drude model (for intraband contributions) and the interband model, both derived from the Kubo formula [50,51]. The optical conductivity σ(ω) refers to the frequency-dependent, which comprises Drude conductivity ( $\sigma_{d}$) and interband transition conductivity ( $\sigma_{in}$). Its expression is given by: $\sigma \left(\omega \right)=\sigma_{d}+Re\left(\sigma_{in}\right)=\frac{e_{2}E_{F}N}{\pi \hbar \left(\gamma -i\omega \right)}+\frac{\pi e^{2}N}{2h}\left[\tanh\left(\frac{2E_{F}+\hbar \omega }{4kT}\right)+\tanh\left(\frac{2E_{F}-\hbar \omega }{4kT}\right)\right].$ In the formula above, *E_F_* stands for the Fermi energy, γ denotes the relation rate, *N* represents the number of layers, *k* is the Boltzmann constant, and *T* is the temperature. The calculation result indicates that the modulation of infrared (IR) absorption stems from two key factors, including the blocking of interband transitions and the enhancement of intraband transitions induced by free carriers.

**Figure 5:**
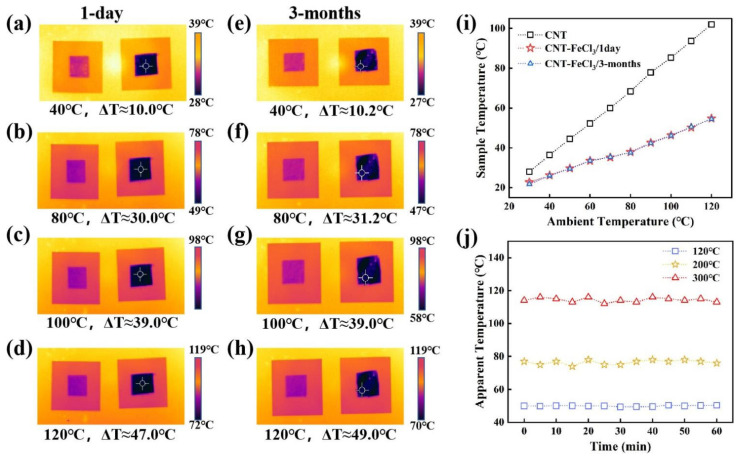
(**a**–**d**) Thermal images of CNT with a lighter color and FeCl_3_-CNT with a darker color at 40 °C, 80 °C, 100 °C, and 120 °C hotplate temperatures. The samples are air-exposed for 1 day. (**e**–**h**) Thermal images of CNT with a lighter color and FeCl_3_-CNT with a darker color at 40 °C, 80 °C, 100 °C, and 120 °C hotplate temperatures. The samples are air-exposed for 3 months. (**i**) Apparent temperature variations of CNT, FeCl_3_-CNT air-exposed for 1 day and 3 months under different background temperatures. (**j**) Apparent temperature stability of FeCl_3_-CNT during 1-h heating at 120 °C, 200 °C, and 300 °C hotplate temperatures.

According to Kirchhoff’s radiation law, for a body in thermal equilibrium, its emissivity (ε) equals its absorptivity (α) at every specific wavelength and corresponding temperature. Emissivity is defined as a measure of how effectively a body emits thermal radiation. Mathematically, this can be expressed as ε(λ,Τ)=α(λ,Τ) [52]. To measure the temperature of the samples, a hot plate is heated within the temperature range of 40–300 °C to simulate diverse background thermal environments. The sample on a glass substrate is placed on the hotplate, and its thermal images are detected by an FLIR T560 thermal camera. The temperature rendered by the camera is referred to as apparent temperature. As shown in Figure 5a–d, the apparent temperatures have significantly decreased after FeCl_3_ intercalation, across the 40–120 °C hotplate temperatures. At 120 °C, the apparent temperature of pristine CNT reaches approximately 103 °C, while that of FeCl_3_-CNT reaches 54 °C, yielding a remarkable temperature difference (∆T) of 49.0 °C between the two materials. Furthermore, correlation analysis between apparent temperatures and background temperature (Figure 5i) yields a coefficient of determination ($R^{2}=1-\frac{SSE}{SST}$) with a value greater than 0.98. Here, SSE is the residual sum of squares and SST is the total sum of squares [53]. This strong linearity indicates that the low thermal radiation characteristics, imparted by FeCl_3_ intercalation, respond to background temperature variations with high predictability and reliability [54], reflecting the exceptional infrared camouflage capability of the intercalated material [55]. After being exposed to air for 3 months, the FeCl_3_-CNT sample exhibited almost the same apparent temperatures as when exposed to air for just 1 day, as illustrated in Figure 5a–h. After continuous heating for 1 h, the apparent temperature of the FeCl_3_-CNT film was 50 ± 0.5 °C, 76 ± 2 °C, and 114 ± 2 °C at background temperatures of 120 °C, 200 °C, and 300 °C, respectively, as shown in Figure 5j. Therefore, FeCl_3_-CNT materials exhibit excellent long-term stability of infrared camouflage performance both in air and a hot environment.

**Figure 6:**
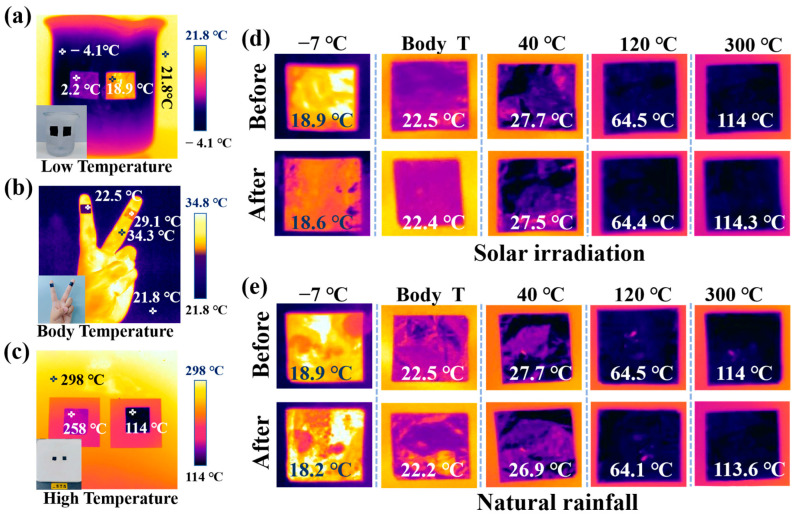
(**a**) Thermal images of pristine CNT (left, 2.2 °C) and FeCl_3_-CNT (right, 18.9 °C) on a beaker at a temperature of −4 °C. (**b**) Thermal images of pristine CNT (middle finger, 29.1 °C) and FeCl_3_-CNT (index finger, 22.5 °C). (**c**) Thermal images of pristine CNT (left, 258 °C) and FeCl_3_-CNT (right, 114 °C) on the hotplate at a temperature of 298 °C. (**d**) Thermal images of FeCl_3_-CNT samples before and after 2 h of solar irradiation. (**e**) Thermal images of FeCl_3_-CNT samples before and after 2 h of natural rainfall.

The thermal camouflage capability of the CNT film before and after FeCl_3_ intercalation was assessed under low, body, and high temperature conditions, as shown in Figure 6a–c. For low-temperature conditions, the samples were attached to a glass cup filled with ice (−4 °C). FeCl_3_-CNT exhibits a measured apparent temperature of 18.9 °C, far nearer to the air temperature (21.8 °C) than the CNT sample with a measured temperature of 2.2 °C (Figure 6a). The two materials exhibit a ∆T of 16.7 °C. For body-temperature tests, the samples were attached to a volunteer’s finger (34.3 °C). The apparent temperature was 22.5 °C in the FeCl_3_-CNT film but 29.1 °C in the CNT (Figure 6b), giving a ∆T of 6.6 °C. For high-temperature conditions, the samples were placed on a hot plate with a constant temperature of 300 °C. The CNT showed a temperature of 258 °C while the FeCl_3_-CNT temperature was only 114 °C (∆T = 144 °C). These results demonstrate that FeCl_3_ intercalation can significantly enhance the thermal camouflage capability of the CNT film across a wide temperature range. To assess fitness for complex application environments, solar irradiation and natural rainfall exposure tests are conducted on the FeCl_3_-CNT film. As presented in Figure 6d,e, across background temperatures of −7 °C, body temperature, 40 °C, 120 °C, and 300 °C, the apparent temperature of the sample shows a variation of no more than 1 °C before and after solar irradiation and natural rainfall exposure for 2 h.

## 3. Conclusions

This study synthesizes FeCl_3_-CNT via a dual-zone vapor deposition method. A suppression of broadband infrared absorption from 72% for CNT to 30% for FeCl_3_-CNT was observed across the wavelength range of 2.5 to 15.2 μm. Under low (−4 °C), body (34.3 °C), and high temperature (300 °C) conditions, compared with CNT film, FeCl_3_-CNT shows enhanced thermal camouflage capability, with the apparent temperature increases by 16.7 °C, and decreases by 6.6 °C and 144 °C, respectively. The thermal camouflage exhibits outstanding stability, with no significant temperature change detected under three conditions, namely, heating at 300 °C for 1 h, and solar irradiation and natural rainfall exposure lasting 2 h. This study demonstrates that intercalation serves as a promising strategy for the design of infrared camouflage materials, which hold significant application potential in the aerospace, military, and wearable device sectors.

## 4. Experimental Section

### 4.1. Material Preparation and FeCl_3_ Intercalation

Multi-walled carbon nanotube (MWCNT) film was synthesized via the floating catalyst chemical vapor deposition (FCCVD) method and purchased from Jiacai Technology. All carbon nanotube materials employed in this work were MWCNTs; for the sake of brevity, the material is designated as simply carbon nanotube (CNT). CNT samples were cut into 1 cm × 1 cm and placed flat on 2 cm × 2 cm glass slides. Then, a weighing paper was laid on the CNT sample, and gentle pressure was applied to secure the CNT’s uniform and flat adhesion to the glass slide. Finally, the samples were dried in a 60 °C vacuum oven for 8 h.

The obtained 1 cm × 1 cm CNT film and 0.3 g anhydrous FeCl_3_ powder (98% Alfa Aesar, Shanghai, China) were placed in separate zones within a quartz tube, and the tube was then sealed. All these operations were carried out in an argon-filled glovebox (<0.01 ppm O_2_/H_2_O) to prevent the anhydrous FeCl_3_ powder from absorbing moisture. Then, the tube was transferred to an intelligent temperature-controlled heating jacket for intercalation at 360 °C for 1.5 h. Once the heating jacket cools down to room temperature, the intercalated CNT film was removed.

### 4.2. Characterization and Measurement

Raman spectroscopy was performed using a continuous-wave laser system (532 nm excitation), with emitted signals collected via a spectrometer coupled to an optical microscope (×50 objective), focusing the laser directly on the samples. Morphology and micro-structure were observed using a Hitachi SU8010 field-emission scanning electron microscope (SEM) operating at 5.0 kV. Chemical compositions and structural properties were analyzed using X-ray photoelectron spectroscopy (XPS) on a Thermo Scientific^TM^ (Waltham, MA, USA) ESCALAB Xi+ spectrometer with monochromatic Al Kα radiation (1486.6 eV, C 1s referenced to 284.8 eV), and X-ray diffraction (XRD) on an Aeris Research benchtop diffractometer with Cu Kα radiation (λ = 1.5406 Å, 2θ = 5°–60° at 0.02° step size, 10 min/scan rate). Fourier transform infrared spectroscopy (FTIR) was performed on a Thermo Scientific™ NicoletTM iS50 (Waltham, MA, USA) spectrometer in attenuated total reflectance (ATR) mode (4000–400 cm^−1^ range, 4 cm^−1^ resolution). Electrical resistivity measurements are based on a van der Pauw Resistivity Measurement Method with a Keithley 2602B Source Meter [56]. The connection configuration between the sample and the electrodes is provided in the Supporting Information (Figure S2). Infrared images were acquired using a FLIR T560 infrared camera (Wilsonville, OR, USA), with the wavelength range of 7.5–14 μm, followed by processing with FLIR Tools 5.7 Research software.

## Data Availability

All data needed to support the conclusions in the paper are presented in the manuscript. Additional data related to this paper may be requested from the corresponding author upon request.

## Supplementary Materials

The following supporting information can be downloaded at: https://www.mdpi.com/article/10.3390/mi17010038/s1, Figure S1. High-resolution SEM images of MWCNT (a) before and (b) after FeCl_3_ intercalation. Figure S2. The sample is intended for electrical property measurements.
