# Supplementary material for: FeCl3-Intercalated Carbon Nanotube Film for Long-Term Infrared Camouflage in Harsh Environments
Source: Micromachines (Basel). 2025 Dec 29;17(1):38. doi: 10.3390/mi17010038 (PMC12843996; doi:10.3390/mi17010038)
Supplement: Supplementary file 1 [file micromachines-17-00038-s001.zip › micromachines-4033316-supplementary.pdf]

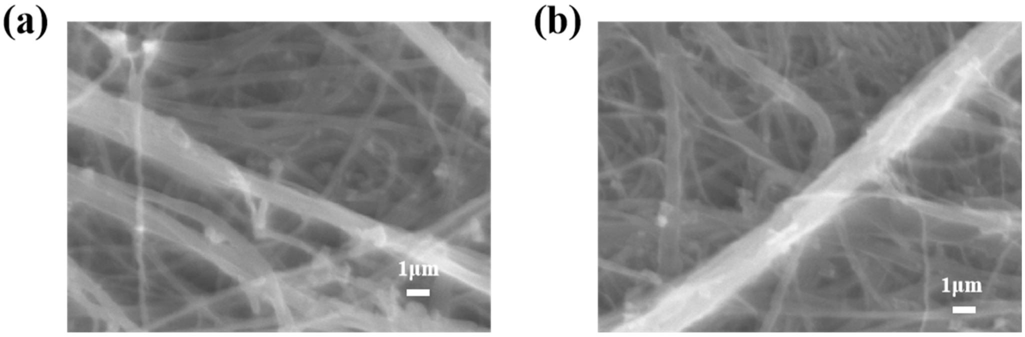

Figure S1. High-resolution SEM images of MWCNT (**a**) before and (**b**) after $FeCl_3$ intercalation.

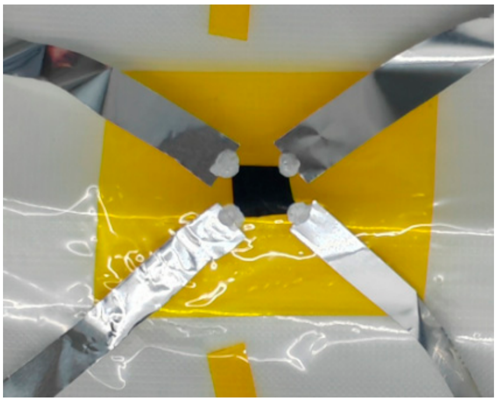

Figure S2. The sample is intended for electrical property measurements.
